# Direct effects of glucagon-like Peptide-1 receptor agonists on mitochondrial function in human-derived in vitro models: A systematic review and meta-analysis

**DOI:** 10.1016/j.metop.2026.100484

**Published:** 2026-07-02

**Authors:** Zoe Lee Greenblatt, Atena Tork, Ivy Cruz, Erik D. Fausak, Huy-an Tran, Mahima Krovidy, Cecilia Giulivi

**Affiliations:** a Department of Molecular Biosciences, School of Veterinary Medicine, University of California Davis, Davis, CA, USA; b MIND Institute, University of California at Davis Medical Center, Sacramento, CA, USA; c University Library, University of California, Davis, CA, 95616, USA

**Keywords:** Glucagon-like peptide-1 receptor agonists, Mitochondrial function, Bioenergetics, Mitochondrial reactive oxygen species, Mitochondrial membrane potential, Systematic review, Meta-analysis

## Abstract

GLP-1 receptor agonists (e.g., semaglutide; GLP-1 RAs) treat Type 2 Diabetes and obesity, mainly by improving glycemic control, suppressing appetite, delaying gastric emptying, and inducing weight loss. Emerging evidence suggests they also directly affect mitochondrial function independently of systemic metabolism. To our knowledge, this is the first systematic review and meta-analysis examining direct mitochondrial effects of GLP-1 RAs in human-derived in vitro models. Of 1547 records identified (1203 screened after deduplication), 17 studies met the criteria, and only 11 contributed to the quantitative synthesis of mitochondrial outcomes. Outcomes included mitochondrial membrane potential (MMP), bioenergetics (ATP-linked oxygen consumption, respiration, ATP production), and mitochondrial reactive oxygen species (MitoROS). Meta-analysis demonstrated significantly improved bioenergetics (SMD = 1.109, 95% CI: 0.556–1.662, P < 0.001) and reduced MitoROS (SMD = −3.489, 95% CI: −6.690 to −0.288, P = 0.034) following GLP-1RA treatment. No significant effect on MMP was observed in the primary analysis (SMD = 0.997, 95% CI: −1.678 to 3.672, P = 0.459), although an exploratory sensitivity analysis excluding statistically identified outlying effect sizes suggested a potential improvement in MMP (SMD = 3.145, 95% CI: 2.147–4.144, P < 0.01); however, this finding should be interpreted cautiously. Overall certainty of the evidence was very low for the primary outcomes due to methodological limitations, substantial heterogeneity, imprecision, and publication bias in some outcomes. Only the MMP sensitivity analysis achieved low-certainty evidence. These findings suggest that GLP-1 RAs may directly promote mitochondrial health, but more rigorous, standardized, and independent studies are needed to confirm their relevance to whole-body physiology.

## Highlights

• GLP-1 RAs were associated with improved mitochondrial bioenergetics in human-derived in vitro models.
• The certainty of the evidence was low due to methodological limitations and publication bias.
• Standardized and independently replicated studies are needed.

## Graphical abstract

**Figure:**
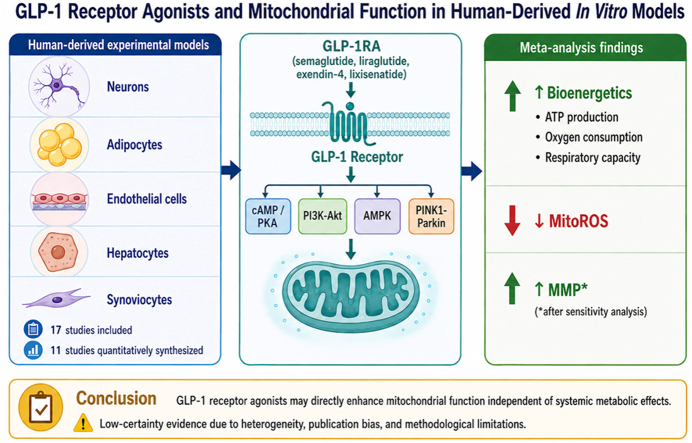

## 1 Introduction

Glucagon-like peptide-1 receptor agonists (GLP-1 RAs), commonly marketed as Ozempic, Zepbound, and Wegovy, have rapidly become central to the management of Type 2 Diabetes Mellitus (T2DM) and obesity [1,2]. Beyond their glucose-lowering effects, GLP-1RAs produce clinically meaningful and sustained reductions in body weight [2]. These effects arise from a combination of appetite suppression, delayed gastric emptying, increased satiety, and modulation of central nervous system pathways that govern energy homeostasis and feeding behavior [3,4]. In particular, GLP-1RAs influence hypothalamic and brainstem networks involved in appetite regulation and may also modulate dopamine-mediated reward circuits, thereby reducing hedonic food intake and food-related preoccupation (“food noise”) [5].

While the systemic metabolic and neurobehavioral benefits of GLP-1 RAs are well documented, their direct effects on mitochondrial biology and cellular energy metabolism remain less well defined. In particular, it is unclear to what extent observed changes in cellular bioenergetics reflect primary GLP-1 receptor-mediated signaling versus secondary adaptations resulting from improved metabolic status.

Weight loss induced by reduced caloric intake directly alters the availability of substrates for mitochondrial oxidative phosphorylation [6,7], the primary process by which cells generate adenosine triphosphate (ATP). Mitochondria play a critical role in maintaining cellular homeostasis, regulating processes such as reactive oxygen species (ROS) production, apoptosis, and metabolic flexibility [[8], [9], [10]]. Disruptions in mitochondrial function are implicated in a wide range of chronic diseases, including metabolic disorders, neurodegeneration, and cardiovascular disease [11]. Importantly, disentangling the direct cellular effects of GLP-1 RAs from their systemic metabolic effects remains challenging in clinical studies. Weight loss, altered nutrient availability, improved insulin sensitivity, and changes in energy expenditure can independently influence mitochondrial function, making it difficult to determine whether observed mitochondrial benefits result directly from GLP-1 receptor activation or secondarily from broader metabolic improvements. Human-derived cellular and tissue models provide an opportunity to examine mitochondrial responses in isolation, minimizing these systemic confounding factors and allowing investigation of direct mitochondrial effects. However, these reductionist systems cannot fully capture the complex interactions among organs, endocrine pathways, and metabolic feedback networks that occur *in vivo*. To address this translational limitation, this review was designed to evaluate mechanistic evidence derived from human-derived cellular, tissue, and ex vivo experimental models while interpreting the findings within the context of their preclinical nature. Accordingly, results were synthesized with consideration of the experimental model used, and findings from in vitro studies were interpreted as mechanistic evidence of direct mitochondrial effects rather than as definitive indicators of organism-level metabolic responses, which are influenced by additional systemic and tissue-specific factors. Therefore, in the context of this review, "direct” refers to mitochondrial effects observed in isolated human-derived cells and tissues in the absence of systemic physiological influences, rather than to direct intracellular interactions with mitochondrial components.

Emerging preclinical evidence suggests that GLP-1RAs may exert direct effects on mitochondrial bioenergetics independent of their systemic metabolic actions [[12], [13], [14], [15], [16], [17], [18], [19], [20], [21], [22], [23], [24], [25]]. GLP-1 receptor activation initiates intracellular signaling through cyclic AMP (cAMP)-dependent pathways, with downstream activation of mediators including protein kinase A (PKA), phosphatidylinositol-3 kinase (PI3K)/Akt, and AMP-activated protein kinase (AMPK), all of which have been implicated in regulating mitochondrial metabolism, oxidative stress responses, and cellular energy homeostasis. Within this broader signaling network, putative downstream effectors such as SIRT1, PGC-1α, STAT3, Epac/Akt, and the PINK1/Parkin mitophagy pathway have been proposed to contribute to mitochondrial biogenesis, respiration, antioxidant defenses, and mitochondrial quality control in human-derived experimental models, although evidence remains limited [[19], [20], [21],^26^]. Notably, the studies informing this framework were conducted in vitro, including one transformed human neuroblastoma cell line (SH-SY5Y) and one primary human endothelial cell model (HUVECs). As such, these pathways are discussed as putative mechanisms that align with the human-derived scope of this review but require validation across additional human cell and tissue systems [21,27].

However, interpretation of the mitochondrial literature remains challenging due to substantial methodological heterogeneity, including differences in cell types, disease models, GLP-1 RA agents, dosing regimens, treatment durations, and mitochondrial outcome measures. While most studies report favorable effects on mitochondrial bioenergetics and oxidative stress, the magnitude and consistency of effects on specific endpoints, particularly mitochondrial membrane potential, vary across experimental contexts.

Although emerging evidence suggests that dual- and multi-receptor incretin agonists may also influence mitochondrial function [25,26], the present review was restricted to single-receptor GLP-1 RAs to reduce mechanistic heterogeneity and maintain a focused scope. Dual GLP-1 Ras were excluded to isolate the direct mitochondrial effects attributable to GLP-1 receptor signaling. Although emerging evidence suggests that GIP receptor activation may influence mitochondrial biogenesis, cellular bioenergetics, and oxidative stress through signaling pathways that partially overlap with those activated by GLP-1 RAs, the relative contribution of each receptor pathway remains incompletely defined. Inclusion of dual agonists would therefore have complicated attribution of observed mitochondrial effects to GLP-1 receptor activation alone. While this approach may exclude potentially relevant mitochondrial data, it was intended to reduce mechanistic confounding rather than introduce selection bias and to preserve the interpretability of conclusions regarding GLP-1 receptor-specific effects.

Despite growing interest in the pleiotropic actions of GLP-1RAs, uncertainty remains regarding their direct effects on mitochondrial function in human-derived experimental systems. To date, no systematic review has specifically synthesized and quantitatively evaluated the direct effects of single-receptor GLP-1RAs on mitochondrial function in human-derived experimental models. Consequently, it remains unclear whether the reported mitochondrial effects are consistent across human-derived systems and which aspects of mitochondrial function are most consistently affected.

Accordingly, this systematic review and meta-analysis addressed the following research question: *In human-derived cellular, tissue, and* ex vivo *experimental models, how does treatment with GLP-1 receptor agonists, compared with appropriate control conditions, affect key measures of mitochondrial function, including mitochondrial membrane potential (MMP), mitochondrial bioenergetics, and mitochondrial reactive oxygen species (MitoROS)?* MMP, cellular bioenergetics, and MitoROS were selected as the primary outcomes because they were the most consistently reported measures of mitochondrial function across the included studies and represent complementary dimensions of mitochondrial biology. Specifically, MMP reflects the integrity of the electrochemical gradient required for ATP synthesis, bioenergetic measures assess mitochondrial energy production, and MitoROS provides an indicator of mitochondrial redox balance and oxidative stress. Together, these outcomes provide an integrated assessment of mitochondrial function and cellular energy metabolism. To answer this question, we systematically synthesized and quantitatively analyzed the available evidence while evaluating methodological quality and certainty of the evidence using structured risk-of-bias and modified GRADE frameworks. Ultimately, this review aims to clarify whether GLP-1RAs exert direct mitochondrial effects independent of systemic metabolic influences and to provide a mechanistic foundation for future studies investigating their potential contribution to cellular energy homeostasis and disease pathophysiology.

## 2 Methods

### 2.1 Search strategy

**Fig. 1:**
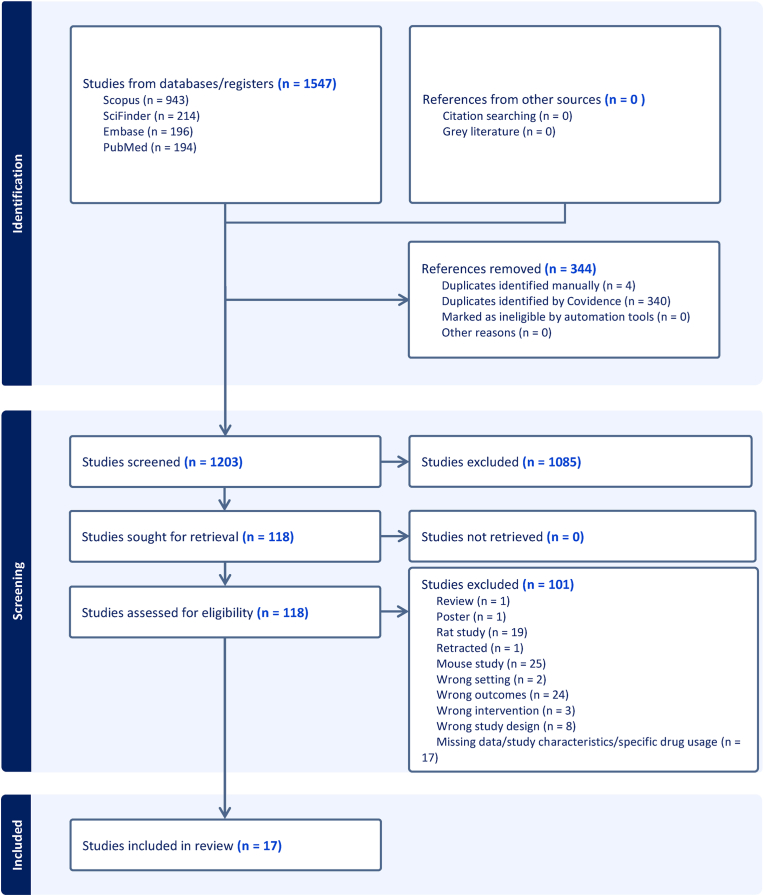
***PRISMA flow diagram of the study selection process.*** Flowchart summarizing the identification, screening, eligibility assessment, and inclusion of studies evaluating the effects of GLP-1 RAs on mitochondrial function in humans. A total of 1547 records were identified through database searches, with 344 duplicates removed prior to screening. Following title/abstract screening and full-text assessment, 17 studies met the inclusion criteria for qualitative synthesis, of which 11 were eligible for quantitative analysis. Reasons for exclusion at the full-text stage included inappropriate study design, outcomes, interventions, or use of non-human models. Detailed search strategy provided in **Supplementary Methods**. Abbreviations: PRISMA, Preferred Reporting Items for Systematic Reviews and Meta-Analyses.

This systematic review and meta-analysis searched PubMed, Scifinder, Embase, and Scopus for studies investigating the effects of GLP-1 RAs on mitochondrial parameters in human-derived cells, tissues, or biological samples (i.e., studies utilizing human-derived experimental models, including primary cells, tissue biopsies, or ex vivo samples). The literature search was conducted in two stages. An initial search was performed on October 4, 2025, and an updated search was conducted on March 31, 2026, prior to final analysis and manuscript preparation to identify newly published studies. Identical database-specific search strategies, eligibility criteria, and screening procedures were applied across both search waves. Records identified during the updated search underwent the same title, abstract, and full-text screening process as records identified during the initial search. Search strategies were adapted for each database using combinations of controlled vocabulary (where applicable), free-text keywords, Boolean operators, and database-specific syntax to identify studies evaluating GLP-1 receptor agonists and mitochondrial function. The complete search strategies, including all database-specific search strings, filters, interfaces, and search dates, are provided in Supplementary Methods in accordance with PRISMA-S recommendations [28,29] accompanied by the PRISMA flow chart (Fig. 1). This systematic review and meta-analysis were prospectively registered in PROSPERO (registration number: CRD420261326658).

### 2.2 Title, abstract, and full text screening

Covidence was used to screen titles and abstracts. Screeners ZLG, AT, MK, HT and CG assessed individual papers based on six main criteria: (i) Written in English; (ii) original research; (iii) (iii) studies utilizing human-derived experimental models (including primary human cells, immortalized human cell lines, human tissue-derived cells, induced pluripotent stem cell (iPSC)-derived models, or ex vivo human tissues or biological samples); (iv) not a review, conference abstract, or patent application; (v) indicated usage of a GLP-1 RA; and (vi) investigated mitochondrial outcomes under physiological conditions or within naturally occurring or experimentally induced disease models relevant to chronic human diseases, including in vitro disease-modeling paradigms (e.g., high glucose, palmitic acid, 6-OHDA, methylglyoxal, or inflammatory stimuli). No restrictions were placed on experimental study design (e.g., randomization, blinding, or use of concurrent controls). Eligible mechanistic in vitro studies were included regardless of these design features, which were subsequently evaluated as part of the modified risk-of-bias assessment rather than used as exclusion criteria.

Papers that met all six inclusion criteria were included and proceeded to full-text review by ZLG, IC, MK, AT, and CG. During full-text review, studies were excluded if they (i) used exclusively animal-derived cells or tissues, (ii) evaluated human-derived cells or tissues only within *in vivo* animal models (e.g., xenograft or transplantation studies), (iii) did not report eligible mitochondrial outcome measures, (iv) were reviews, conference abstracts, editorials, or other non-original research articles, (v) were not published in English, (vi) provided insufficient data to determine study characteristics, intervention details, or outcome measures, (vii) had been retracted, or (viii) did not meet the predefined eligibility criteria for study design, intervention, experimental setting, or outcomes. Studies reporting only non-mitochondrial oxidative stress markers (e.g., total intracellular ROS, malondialdehyde, or antioxidant enzyme activity) without a direct mitochondrial outcome were also excluded (e.g., Ref. [24]).

Seventeen studies met the eligibility criteria and proceeded to data extraction. Following the extraction and application of outcome-specific eligibility criteria, 11 studies provided sufficient quantitative data for inclusion in the meta-analysis (Table 1).

**Table 1:** Characteristics of included in-vitro studies investigating GLP-1 receptor agonists and mitochondrial outcomes^a^.

FIRST AUTHOR, YEAR	DISEASE/EXPERIMENTAL CONTEXT	GLP-1 RECEPTOR AGONIST	DOSE	CELL MODEL (KEY FEATURES)	DONOR AGE∗∗	SEX	MITOCHONDRIAL OUTCOME	TISSUE ORIGIN
**DU, 2019** [23]	Rheumatoid arthritis inflammation	Lixisenatide	10 nM and 20 nM	Human rheumatoid arthritis fibroblast-like synoviocytes	50.1 ± 5.2 y	F/M	Reduced inflammatory and oxidative stress responses	Synovial tissue
**JO, 2021** [15]	Palmitic acid–induced oxidative stress and brain insulin resistance	Exendin-4	10 nM	SH-SY5Y neuroblastoma cells	NA	NA	Improved mitochondrial function and neurite outgrowth	Brain
**LIU, 2022** [16]	Parkinson's disease/6-OHDA toxicity	Liraglutide and Semaglutide	10 nM	SH-SY5Y neuroblastoma cells	NA	NA	Enhanced autophagy and reduced oxidative stress	Brain
**PANAGAKI, 2023** [18]	Parkinson's disease/α-synuclein pathology	Liraglutide	100 nM	LUHMES dopaminergic-like neurons	NA	NA	Restored ER-mitochondrial axis and promoted cell survival	Brain
**PANDEY, 2023** [19]	High glucose–induced mitochondrial dysfunction	Exendin-4	20 nM	SH-SY5Y neuroblastoma cells	NA	NA	Improved mitochondrial function and reduced ROS via GLP-1R/Epac/Akt signaling	Brain
**VAITTINEN, 2023** [22]	Mitochondrial dysfunction in obesity-related adipocytes	Liraglutide and TNF alpha	100 nM	Human Simpson Golabi Behmel syndrome (SGBS) pre-adipocytes	NA	NA	Improved mitochondrial respiration and bioenergetics	Adipose tissue
**YU, 2019** [20]	Non-alcoholic steatohepatitis (NASH)	Liraglutide	100 nM	HEPG2 human hepatocytes	NA	NA	Activated mitophagy and reduced inflammasome activation	Liver
**ZHANG, 2022** [21]	High glucose–induced endothelial dysfunction	Liraglutide	20 nM	Human umbilical vein endothelial cells (HUVECs)	NA	NA	Inhibited excessive PINK1/Parkin-dependent mitophagy	Vascular endothelium
**IGOILLO-ESTEVE, 2020** [13]	Friedreich ataxia	Exenatide	500 nM	Human iPSC-derived sensory neurons from Friedreich ataxia patients	NR	NR	Increased frataxin expression and improved mitochondrial function	Peripheral nervous system (sensory neurons)
**GORALSKA, 2017** [12]	Adipocyte bioenergetic dysfunction	Exendin-4	100 nM	CHUB-S7 human subcutaneous pre-adipocytes	NA	NA	Stimulated mitochondrial bioenergetics	Adipose tissue
**QI, 2022** [27]	Methylglyoxal-induced neuronal toxicity	Liraglutide	100 nM	SH-SY5Y neuroblastoma cells	NA	NA	Reduced oxidative stress and improved cellular energy metabolism	Brain

a Eleven included studies used human-derived in vitro cell models. ∗∗NA, not applicable (established or immortalized cell lines for which donor characteristics are not applicable); NR, for donor-derived human models in which donor characteristics were incompletely reported. Mitochondrial outcomes were grouped to align with quantitative synthesis and GRADE assessment (Table 3).

### 2.3 Data extraction

Data extraction was independently performed by ZLG, AT, and IC using Microsoft Excel and Notability. Extracted variables included study characteristics (author and publication year), disease or experimental context, GLP-1 receptor agonist, treatment dose, experimental model (species, tissue or cell type, and tissue of origin), donor characteristics (age and biological sex, where applicable and reported), mitochondrial outcome measures, and quantitative data required for meta-analysis (sample size, mean, and standard deviation for treatment and control groups). For immortalized cell lines and other experimental models in which donor demographic information was not applicable or not reported, age and biological sex were recorded as not available (NA). If outcome data were reported as mean ± standard error of the mean (SEM), SEM values were converted to standard deviations (SD) using the formula SD = SEM × √n, where n represented the corresponding reported sample size for that experimental group. This conversion assumed that the published SEM values were calculated using the reported group sample sizes. If numerical data were not presented in tables, reviewers independently digitized graphical data using Notability to estimate means and SDs for quantitative synthesis. Each reviewer maintained an independent data extraction sheet.

For studies reporting multiple treatment durations, separate effect sizes were extracted and analyzed independently. In Vaittinen et al. (2023) [22], short-term (ST) liraglutide treatment was defined as 24-h exposure, whereas long-term (LT) treatment was defined as continuous exposure during adipocyte differentiation and maturation (Day 0–15). When essential quantitative data required for eligibility assessment or inclusion in the meta-analysis were unavailable, corresponding authors were contacted by email to request the missing information. Nine corresponding authors were contacted by email. Three responded, although only one provided sufficient information for study inclusion. Consequently, eight studies were excluded because the required data could not be obtained despite reasonable attempts to contact the corresponding authors, or because the information provided was insufficient to determine study eligibility or to permit quantitative synthesis (no response, n = 6; insufficient information, n = 2). The most commonly missing information included participant age range, sex distribution (particularly for control participants), and the specific GLP-1 receptor agonist used.

Potential duplicate or overlapping datasets were assessed by comparing author lists, institutions, publication years, experimental models, cell lines, or tissue sources, treatment conditions, and reported outcome data. When multiple publications appeared to be based on the same experimental dataset, the study that provided the most complete and relevant data was retained. No duplicate or overlapping datasets meeting these criteria were identified among the included studies.

### 2.4 Modified risk of bias (RoB) tool for in-vitro mechanistic studies

Because the eligible studies primarily utilized human-derived cells, tissues, and ex vivo experimental models rather than clinical interventions in living participants, risk of bias was assessed using a modified framework designed for mechanistic in vitro studies. This approach was adapted from the SYRCLE risk-of-bias tool for preclinical research and informed by established methodological principles [30]. Because no universally accepted and validated risk-of-bias instrument currently exists for human-derived in vitro mechanistic studies, we employed a domain-based framework adapted from the SYRCLE risk-of-bias tool and related methodological recommendations for preclinical evidence synthesis [31,32]. The adapted framework retained the domain-based structure of SYRCLE (selection, performance, detection, attrition, and reporting bias) but modified the signaling questions and judgment criteria to reflect features relevant to human-derived mechanistic in vitro studies, including consistency of experimental conditions, objective outcome assessment, completeness of replicate reporting, and mechanistic/translational validity (e.g., receptor specificity, physiological exposure conditions, and model relevance), rather than domains specific to animal intervention studies such as housing or animal allocation procedures. ARRIVE principles were not used as a risk-of-bias assessment tool; rather, they informed evaluation of reporting quality and methodological transparency, which were considered separately from risk-of-bias domains during study appraisal. Although ARRIVE was originally developed for animal research and has not been validated for in vitro studies, its general principles of reporting quality and methodological transparency were considered applicable to mechanistic experimental research. This use was intended solely to inform qualitative assessment of reporting completeness and not to contribute to risk-of-bias judgments.

Selection bias was assessed by examining whether experimental treatment conditions were assigned at random and whether baseline experimental conditions were comparable across treatment groups. Comparability was evaluated based on the use of the same cell line or donor source, passage number (where reported), seeding density, culture conditions (including media and incubation conditions), and identical pre-treatment handling across experimental groups. Studies explicitly reporting random allocation were rated as low risk; those describing allocation procedures without randomization were rated as moderate risk; and those providing no description of allocation were rated as high risk.

Performance bias was evaluated by assessing whether experimental conditions were applied consistently across treatment groups and whether investigators were blinded. Studies that reported blinding or fully automated experimental procedures were rated as low risk. Studies describing standardized protocols without blinding were rated as moderate risk, while studies with unclear experimental handling or variable conditions were rated as high risk.

Detection bias was assessed by evaluating whether outcome assessors were blinded and whether outcome measures were obtained using objective, quantitative methodologies. Studies employing blinded outcome assessment or standardized quantitative assays (e.g., Seahorse extracellular flux analysis, fluorescence-based mitochondrial assays, or other instrument-based quantitative measurements) with clearly described analytical procedures were rated as low risk. Studies reporting objective quantitative outcomes without blinding or with limited description of analytical procedures were rated as moderate risk, whereas studies relying primarily on subjective, observer-dependent, or semi-quantitative outcome measures were rated as high risk.

Attrition bias was evaluated by examining the completeness of outcome data, including the reporting of biological replicates and the justification for any exclusions. Studies clearly reporting replicate numbers and exclusions were rated as low risk. Studies reporting replicate numbers without clear justification for exclusions were rated as moderate risk, and studies with missing data or selective reporting of replicates were rated as high risk.

Reporting bias was assessed based on evidence of selective or incomplete outcome reporting. Studies that reported all mitochondrial outcomes described in the Methods, provided complete quantitative data, or referenced a predefined protocol were rated as low risk. Studies reporting multiple outcomes without preregistration or with minor limitations in reporting completeness were rated as moderate risk. Studies demonstrating evidence of selective outcome reporting, incomplete reporting of prespecified outcomes, or omission of quantitative data necessary for interpretation were rated as high risk.

Other sources of bias specific to mechanistic in vitro research were also considered. These included reliance on a single experimental model, use of GLP-1 receptor agonist concentrations substantially exceeding clinically relevant circulating exposure, and absence of GLP-1 receptor antagonism or genetic knockdown to confirm receptor specificity. Because no universally accepted threshold for supraphysiological exposure exists in mechanistic in vitro studies, concentrations were evaluated in the context of the study objective, exposure duration, and accompanying mechanistic validation rather than according to a single predefined cutoff. Studies employing multiple experimental models with receptor validation were rated as low risk. Studies that used a single experimental model and/or lacked receptor-specificity controls were generally rated as moderate risk. High-risk ratings in the mechanistic/translational validity domain required multiple concerns regarding model relevance, mechanistic specificity, and translational applicability, rather than the presence of any single limitation.

Using this modified framework, none of the included studies were judged to be at low risk of bias across all domains. Most studies (10/11) were rated as having an overall moderate risk of bias, primarily due to incomplete reporting of methodological features such as randomization, blinding, and preregistration, rather than clear evidence of systematic bias (Table 2). One study was rated as having an overall high risk of bias because multiple methodological concerns affecting mechanistic and translational validity were identified. Risk-of-bias assessments were used to inform the certainty of the evidence through the modified GRADE framework rather than as exclusion criteria for quantitative synthesis, consistent with established systematic review methodology.

**Table 2:** Modified risk of bias assessment of included in-vitro studies^a^.

Study (Author, Year)	Selection	Performance	Detection	Attrition	Reporting	Mechanistic/Translational Validity	Overall RoB
Du et al., 2019 [23]	Moderate	Moderate	Moderate	Moderate	Moderate	Moderate	**Moderate**
Zhang et al., 2022 [21]	Moderate	Moderate	Low	Moderate	Moderate	Moderate	**Moderate**
Panagaki et al., 2023 [18]	Moderate	Moderate	Low	Moderate	Moderate	Moderate	**Moderate**
Vaittinen et al., 2023 [22]	Moderate	Moderate	Low	Low	Moderate	Moderate	**Moderate (lowest)**
Yu et al., 2019 [20]	Moderate	Moderate	Moderate	Moderate	High	Moderate	**Moderate**
Jo et al., 2021 [15]	Moderate	Moderate	Low	Moderate	Moderate	Moderate	**Moderate**
Liu et al., 2022 [16]	Moderate	Moderate	Moderate	Moderate	Moderate	Moderate	**Moderate**
Pandey et al., 2023 [19]	Moderate	Moderate	Low	Moderate	Moderate	Moderate	**Moderate**
Góralska et al., 2017 [12]	Moderate	Moderate	Low	Moderate	Moderate	Moderate	**Moderate**
Igoillo-Esteve et al., 2020 [13]	Moderate	Moderate	Low	Moderate	Moderate	Moderate	**Moderate**
Qi et al., 2022 [27]	Moderate	Moderate	Moderate	Moderate	Moderate	High	**High**

a Risk of bias was assessed using a modified framework for in-vitro mechanistic studies adapted from SYRCLE and ARRIVE guidelines. Domains were rated as Low, Moderate, or High risk of bias. Overall risk of bias reflects the highest risk rating across domains, accounting for cumulative methodological limitations. No included study was rated as low risk of bias across all domains. Mechanistic/translational validity ratings reflected an overall assessment of model relevance, receptor specificity, physiological exposure conditions, and translational applicability. Individual concerns, including the use of supraphysiological concentrations of GLP-1 RAs, were considered in the broader context of the study design and did not automatically result in a high-risk rating.

### 2.5 GRADE assessment of mitochondrial outcomes

Consistent with established systematic review methodology, study-level risk-of-bias assessments were used to inform interpretation of the evidence and certainty ratings rather than as exclusion criteria for quantitative synthesis. Excluding studies solely on the basis of risk-of-bias ratings was not prespecified and may itself introduce bias. Moreover, because only one included study was judged to be at an overall high risk of bias and the number of eligible studies contributing to each mitochondrial outcome was small, excluding studies on the basis of risk of bias would have substantially reduced the available evidence and limited the feasibility of meaningful quantitative synthesis. Accordingly, all eligible studies were included in the meta-analysis, and risk-of-bias assessments were incorporated into a modified GRADE approach to determine the overall certainty of the evidence.

GRADE certainty ratings were based on quantitative syntheses including all eligible studies, regardless of risk-of-bias classification, using a modified GRADE framework for mechanistic in vitro studies [[33], [34], [35]]. All outcomes were assigned an initial rating of low certainty because the evidence was derived from human-derived cellular, tissue, and ex vivo experimental models rather than clinical studies. Inconsistency was assessed using the I^2^ statistic and the direction of effect across studies. High statistical heterogeneity (I^2^ > 50%) and inconsistent effect direction were considered evidence of serious inconsistency. Imprecision was evaluated based on confidence interval width and exclusion of the null effect. Publication bias downgrades were applied only when supported by formal statistical testing.

Although GRADE permits upgrading for large effect sizes under specific circumstances, no upgrading was applied in the present review because the observed effect estimates were accompanied by important methodological limitations, including substantial heterogeneity, moderate-to-high risk of bias, small evidence bases, indirectness inherent to mechanistic in vitro models, and evidence of publication bias for some outcomes. Consequently, large standardized mean differences were not considered sufficient, in isolation, to increase certainty in the evidence.

### 2.6 Mitochondrial outcomes

All outcomes included in the quantitative synthesis were derived from human-derived experimental models investigating mitochondrial function following GLP-1 RAs treatment. Mitochondrial bioenergetic outcomes included quantitative measures reflecting mitochondrial energy production, including ATP-linked oxygen consumption rate (OCR), basal respiration, maximal respiration, and ATP production. Where available, OCR-derived parameters corresponded to standardized measurements obtained using extracellular flux (Seahorse) analysis according to the definitions reported in the original studies. For studies using alternative methodologies (e.g., ATP quantification assays or other validated assessments of mitochondrial bioenergetics), conceptually equivalent measures of mitochondrial energy production were extracted and categorized according to the predefined outcome domains. Outcome definitions reported by the original investigators were retained, and studies were grouped by biological construct rather than by assay platform. Other mitochondrial endpoints reported by individual studies were summarized qualitatively but were not included in meta-analysis because insufficient data were available for quantitative pooling.

#### 2.6.1 Statistical analysis

Meta-analyses were performed using MedCalc® Statistical Software version 23.5.2 (MedCalc Software Ltd., Ostend, Belgium). Continuous outcomes were pooled using standardized mean differences (SMDs) with corresponding standard errors (SEs) and 95% confidence intervals (CIs), as mitochondrial outcomes were measured using different assays and reporting scales across studies. For the bioenergetics meta-analysis, multiple related bioenergetic endpoints (e.g., ATP-linked oxygen consumption rate, basal respiration, maximal respiration, and ATP production) reported within the same study were included as separate effect sizes.

Because within-study correlation coefficients among multiple bioenergetic outcomes were not reported, a multivariate meta-analysis could not be performed. Consequently, conceptually related bioenergetic measures (e.g., ATP-linked oxygen consumption rate, basal respiration, maximal respiration, and ATP production) were analyzed as separate effect sizes. This approach assumes statistical independence among outcomes and may overestimate precision when multiple correlated measures are included in the same study. Accordingly, this limitation was considered in interpreting the findings and contributed to downgrading the certainty of the evidence in the modified GRADE assessment.

Random-effects models were used for all analyses to account for expected methodological and biological heterogeneity among studies, including differences in disease state, tissue or cell type, GLP-1 receptor agonist, treatment duration, and experimental methodology. Statistical significance was defined as P < 0.05.

Heterogeneity was assessed using Cochran's Q statistic and the I^2^ statistic. I^2^ values of approximately 25%, 50%, and 75% were interpreted as low, moderate, and high heterogeneity, respectively. Where substantial heterogeneity was observed, subgroup and sensitivity analyses were conducted when sufficient data were available. When substantial heterogeneity was observed, outlier diagnostics were performed using a robust fit (Cauchy) procedure in JMP v. 19.1.2 to identify statistically influential outlying effect sizes. Sensitivity analyses were then conducted after excluding these statistically identified outliers, rather than based on visual inspection of forest plots. Separate meta-analyses were performed for mitochondrial bioenergetics, mitochondrial membrane potential (MMP), and mitochondrial reactive oxygen species (MitoROS). For bioenergetic outcomes, the ATP-linked oxygen consumption rate (OCR), basal respiration, maximal respiration, and ATP production were pooled into a single category. Exploratory subgroup analyses were performed after study selection, when sufficient data were available, to investigate potential sources of heterogeneity. These included analyses restricted to adipose tissue-derived studies and, where data permitted, stratification by GLP-1 receptor agonist type. Because of the limited number of eligible studies, these subgroup analyses were considered exploratory and interpreted cautiously. Sensitivity analyses were performed by excluding individual studies identified as potential sources of heterogeneity.

Potential publication bias was evaluated using funnel plots, Egger's regression test, and Begg's rank correlation test (Supplementary Fig. 1). Although Egger's, Begg's, and funnel plots were calculated for completeness, formal inferences regarding publication bias were considered unreliable when fewer than 10 studies were available. As such, given the limited number of studies for several outcomes, publication bias was interpreted with caution.

The certainty of evidence for each pooled outcome was assessed using a modified GRADE framework adapted for preclinical in vitro studies (Table 3). All outcomes were assigned an initial rating of low certainty due to the indirect nature of evidence derived from human-derived cells, tissues, and ex vivo experimental models. Certainty was downgraded based on risk of bias, inconsistency, imprecision, and publication bias. Evidence was not upgraded for large effect sizes because of the mechanistic nature of the included studies and their limited clinical translatability.

**Table 3:** GRADE evidence profile of selected mitochondrial outcomes (sensitivity analysis)^a^.

Outcome	No. of studies (n)	Effect (SMD ± SE)	Overall Modified Risk of Bias	Inconsistency	Indirectness	Imprecision (95% CI)	Publication Bias	Overall Certainty Of Evidence	Justification
**Bioenergetics**	**6**	1.109 ± 0.281 (P < 0.001)	5 with Moderate 1 with high	Serious I^2^ = 71.22%; P = <0.0001	Not serious	Not serious (0.556 to 1.662)	Borderline concern (tests performed but interpreted cautiously because <10 studies) (Egger's P = 0.0002; Begg's P < 0.0001)	**Very low**	Downgraded for substantial heterogeneity and evidence of publication bias. Multiple related bioenergetic outcomes from individual studies contributed separate effect sizes, potentially inflating precision
**Bioenergetics: Adipose Only**	**2**	1.394 ± 0.343 (P < 0.001)	All with Moderate	Serious I^2^ = 63.76%; P = 0.0006	Serious (n = 2 studies)	Serious (0.713 to 2.075)	Unable to assess reliably (Egger's P < 0.0001; Begg's P = 0.0006)	**Very Low**	Downgraded for heterogeneity, limited tissue specificity, small evidence base, and publication bias.
**MitoROS**	**3**	−3.489 ± 1.551 (P = 0.034)	2 with Moderate; one with High	Serious I^2^ = 82.40%; P = 0.0034	Not serious	Serious (−6.690 to −0.288)	Unable to assess reliably (Egger's P = 0.0616; Begg's P = 0.1172)	**Very low**	Downgraded for substantial heterogeneity and imprecision
**MMP**	**5**	0.997 ± 1.336 (P = 0.459)	All with Moderate	Serious I^2^ = 89.98%; P < 0.0001	Not serious	Serious (−1.678 to 3.672)	Unable to assess reliably (Egger's P = 0.2775; Begg's P = 0.8806)	**Very low**	Downgraded for extreme heterogeneity and imprecision
**MMP without Liu 2022**	**4**	3.145 ± 0.495 (P < 0.01)	All with Moderate	Not serious I^2^ = 19.10%; P = 0.23930	Not serious	Not serious (2.147 to 4.144)	Unable to assess reliably (Egger's P = 0.0645; Begg's P = 0.0500)	**Low**	Sensitivity analysis demonstrated consistent effects with low heterogeneity and relatively precise estimates; certainty remained low because evidence originated from preclinical in vitro studies

a Certainty of evidence was assessed using a modified GRADE framework adapted for preclinical in vitro studies. All outcomes were assigned an initial rating of low certainty due to the indirect nature of evidence derived from human-derived cells, tissues, and ex vivo experimental models. Certainty was downgraded based on risk of bias, inconsistency (heterogeneity), imprecision, and publication bias. Evidence was not upgraded for large effect sizes due to the mechanistic nature of the models, potential effect inflation, and limited translational applicability. Borderline publication-bias test results (e.g., P values approximately equal to 0.05) were classified as "possible concern” rather than as definitive evidence of publication bias. Formal downgrading for publication bias was reserved for outcomes demonstrating clear statistical evidence of publication bias across multiple tests.

## 3 Results

### 3.1 Search outcomes and data extraction

A total of 1547 records were identified through searches of PubMed, SciFinder, Embase, and Scopus conducted on October 4, 2025, and March 31, 2026. Following import into Covidence, 344 duplicate records were removed, leaving 1203 studies for title and abstract screening. Of these, 1085 records were excluded because they were not relevant to the effects of GLP-1 RAs on mitochondrial function. Full-text review was conducted for 118 studies, resulting in the exclusion of 101 studies. Seventeen studies met the eligibility criteria and proceeded to data extraction (Fig. 1).

During data extraction, several outcomes were identified, including mitochondrial membrane potential (MMP), bioenergetics, lactate dehydrogenase (LDH), lactate release, reactive oxygen species (ROS), and mitochondrial reactive oxygen species (MitoROS). Quantitative synthesis was restricted to outcomes that directly assessed mitochondrial function and were reported by at least two independent studies (see below). Although ROS measurements have been reported by multiple studies, most have assessed total intracellular ROS rather than mitochondria-specific ROS. Therefore, only MitoROS outcomes were included in the quantitative synthesis. LDH outcomes were excluded because the release of this enzyme is a marker of plasma membrane integrity rather than mitochondrial function. Lactate release was reported in only one study [27] and was therefore not included in the quantitative synthesis. In Qi et al. (2022), liraglutide treatment of methylglyoxal-exposed SH-SY5Y cells did not significantly alter lactate levels compared with untreated methylglyoxal-exposed cells (0.172 ± 0.0875 vs. 0.176 ± 0.055 by NMR), despite the authors reporting broader metabolomic evidence consistent with promoted oxidative phosphorylation and inhibited glycolysis. Consequently, the final meta-analysis focused on three mitochondrial outcomes: bioenergetics, MMP, and MitoROS.

Papers providing essential quantitative data on the impact of GLP-1 RAs on mitochondrial function were eligible for inclusion in the quantitative synthesis (n = 11; Table 1). These studies evaluated mitochondrial function across a range of human-derived experimental models, including adipocytes, neuronal cells (including iPSC-derived sensory neurons), hepatocytes, endothelial cells, and synoviocytes, and investigated multiple GLP-1 RAs, most commonly liraglutide and exendin-4 (Table 1). Across studies, GLP-1 RA-treated cells were generally compared with untreated control cells or disease-model controls exposed to the relevant experimental stressor (e.g., high glucose, palmitic acid, TNF-α, methylglyoxal, α-synuclein, or 6-OHDA) in the absence of GLP-1 receptor agonist treatment.

Quantitative synthesis included 24 bioenergetic effect sizes derived from 6 studies, based on measures such as ATP production, ATP-linked oxygen consumption rate, basal respiration, and maximal respiration, as well as 3 effect sizes for MitoROS and 7 for MMP. Most included studies were judged to have a moderate risk of bias, primarily due to limited reporting of randomization, blinding, and methodological safeguards, while four studies were classified as high risk of bias (Table 2). Overall certainty of the evidence, assessed using a modified GRADE framework, ranged from low to very low for mitochondrial outcomes due to methodological limitations, heterogeneity, small sample sizes, and evidence of publication bias (Table 3).

### 3.2 Bioenergetic outcomes

**Fig. 2:**
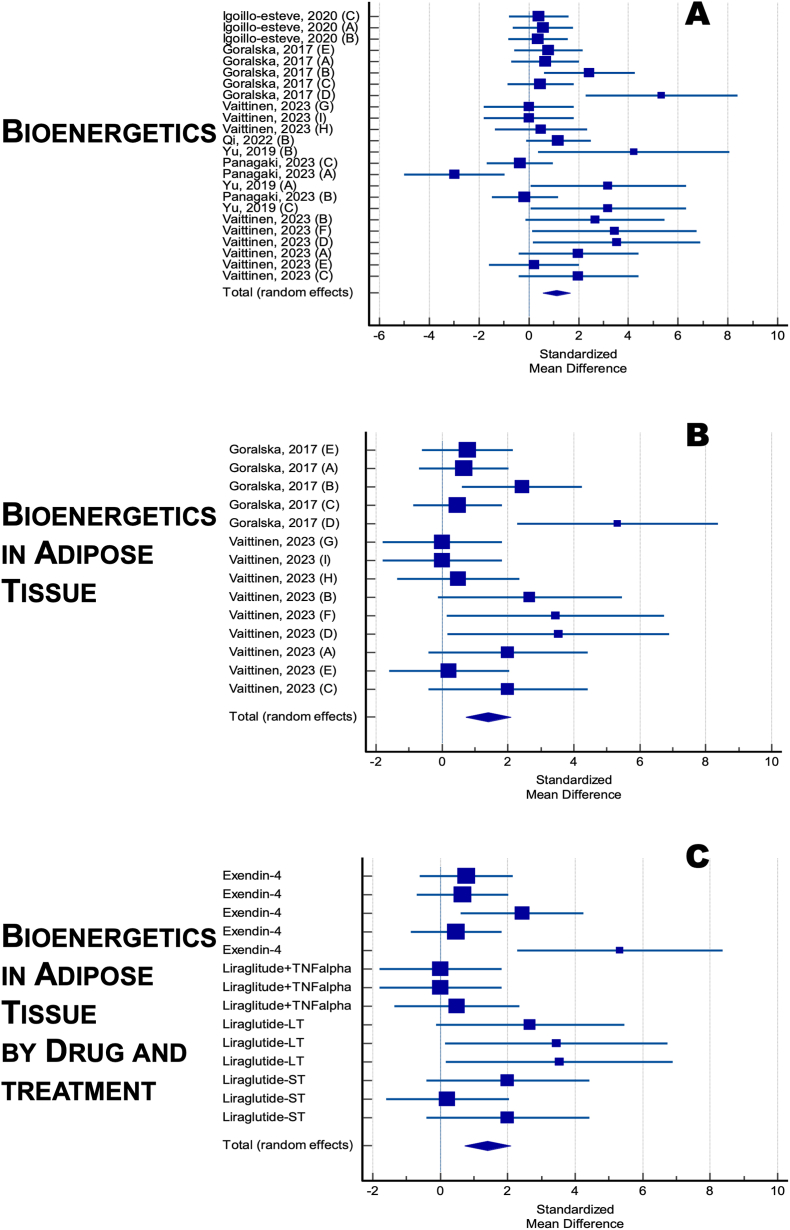
***Forest plots evaluating the effects of GLP-1 receptor agonists on bioenergetic outcomes.*** (A) Forest plot of standardized mean differences for bioenergetic outcomes, including ATP-linked oxygen consumption, basal respiration, and maximal respiration, comparing GLP-1 receptor agonist treatment with control conditions. (B) Forest plot of standardized mean differences for bioenergetic outcomes restricted to adipose tissue studies. (C) Forest plot of standardized mean differences for adipose tissue bioenergetic outcomes stratified by GLP-1 receptor agonist type and treatment (ST and LT = short and long term treatments). Squares represent individual study effect sizes weighted by study variance, horizontal lines indicate 95% confidence intervals, and diamonds represent pooled random-effects estimates. Corresponding numerical meta-analysis results are shown in Supplementary Table 1–3. Letter suffixes denote distinct effect sizes derived from the same study. Multiple effect sizes were included when studies reported separate mitochondrial outcomes, treatment conditions, agonist doses, or cell models that could be analyzed independently. ST = short-term liraglutide treatment (24 h); LT = long-term liraglutide treatment (Day 0–15). Letter suffixes denote distinct effect sizes extracted from the same study based on different mitochondrial outcomes, treatment conditions, doses, or experimental models.

A total of six studies contributed bioenergetic outcomes to the quantitative synthesis (Fig. 2A). Several studies reported multiple eligible bioenergetic endpoints (including measures of ATP-linked oxygen consumption, basal respiration, maximal respiration, and ATP production), resulting in multiple effect sizes per study. In particular, Vaittinen et al. (2023) contributed the largest number of effect sizes because multiple bioenergetic parameters were reported across experimental conditions. Overall, GLP-1 RA treatment was associated with significantly improved mitochondrial bioenergetic function compared with control conditions (SMD = 1.109 ± 0.281, 95% CI: 0.556–1.662, P < 0.001; Supplementary Table 1). However, substantial between-study heterogeneity was observed (I^2^ = 71.22%, P < 0.0001; SFig. 1A).

As prespecified in the statistical analysis plan, a subgroup analysis of adipose tissue-derived studies was performed (Fig. 2B). This analysis was undertaken because adipose tissue represented the most frequently studied tissue type and was considered biologically relevant to the metabolic actions of GLP-1 RAs. In this subgroup, GLP-1 RA treatment remained associated with significantly improved bioenergetic outcomes (SMD = 1.394 ± 0.343, 95% CI: 0.713–2.075, P < 0.001; Supplementary Table 2), although moderate-to-high heterogeneity persisted (I^2^ = 63.76%, P = 0.0006; SFig. 1B).

Further stratification by GLP-1 RA type within adipose tissue studies demonstrated generally positive effects across treatment groups (Fig. 2C; Supplementary Table 3). However, interpretation of differences between agents should be approached with caution because agent-specific comparisons were confounded by between-study differences in experimental design, cell models, and assay conditions, with the majority of effect sizes attributable to a single study (SFig. 1C). Despite these limitations, the overall direction of effect consistently favored GLP-1 RA treatment, suggesting improved mitochondrial bioenergetic function following exposure to these agents.

### 3.3 Mitochondrial reactive oxygen species (MitoROS)

**Fig. 3:**
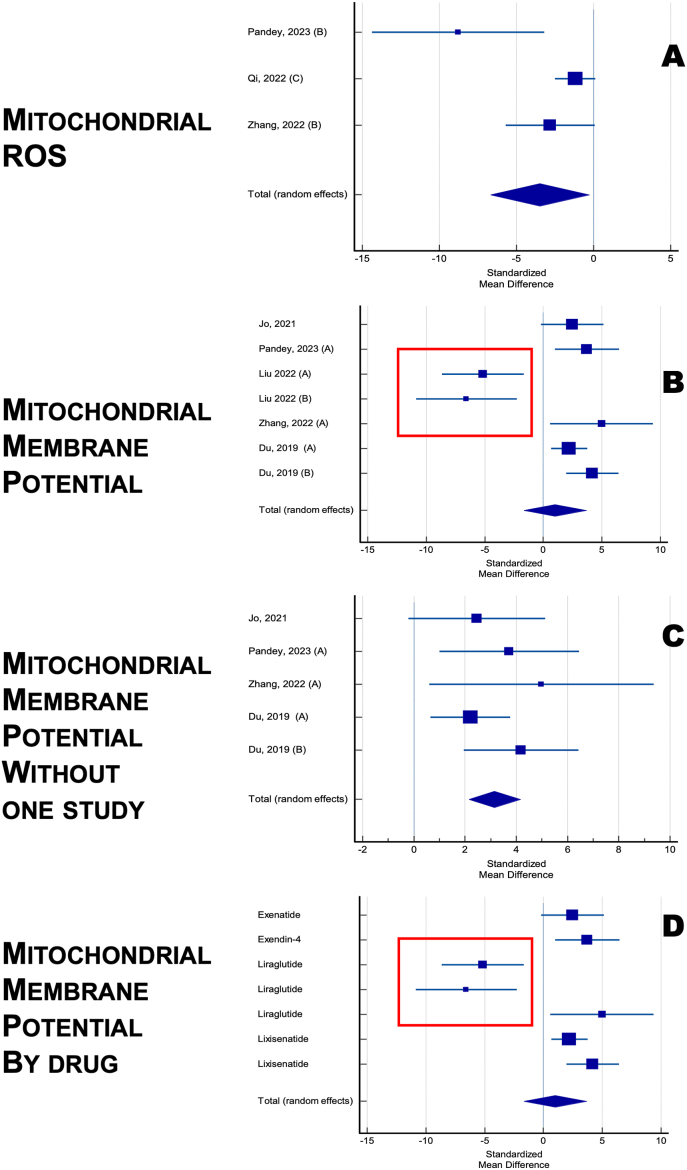
***Forest plots evaluating the effects of GLP-1 receptor agonists on mitochondrial ROS (MitoROS) and mitochondrial membrane potential (MMP) outcomes.*** (A) Forest plot of standardized mean differences for MitoROS outcomes comparing GLP-1 receptor agonist treatment with control conditions. (B) Forest plot of standardized mean differences for MMP outcomes comparing GLP-1 receptor agonist treatment with control conditions. Red boxes are provided for illustrative purposes to identify effect sizes that are subsequently classified as statistical outliers. Outlier status was determined using a robust fit (Cauchy) diagnostic procedure and not by visual inspection of the forest plots. (C) Forest plot of standardized mean differences for MMP outcomes excluding Liu et al. (2022). (D) Forest plot of standardized mean differences for MMP outcomes stratified by GLP-1 receptor agonist type. Drug labels correspond to the terminology used in the original studies and identify individual study-level effect sizes rather than pooled pharmacological categories. Exenatide represents the synthetic form of exendin-4 and is pharmacologically equivalent. Red boxes are provided for illustrative purposes to identify effect sizes that are subsequently classified as statistical outliers. Outlier status was determined using a robust fit (Cauchy) diagnostic procedure and not by visual inspection of the forest plots. Squares represent individual study effect sizes weighted by study variance, horizontal lines indicate 95% confidence intervals, and diamonds represent pooled random-effects estimates. Corresponding numerical meta-analysis results are shown in Supplementary Table 4–7. Letter suffixes denote distinct effect sizes derived from the same study. Multiple effect sizes were included when studies reported separate mitochondrial outcomes, treatment conditions, agonist doses, or cell models that could be analyzed independently. (For interpretation of the references to colour in this figure legend, the reader is referred to the Web version of this article.)

Three studies contributed data to the MitoROS meta-analysis (Fig. 3A). Overall, GLP-1 RA treatment was associated with a significant reduction in mitochondrial reactive oxygen species production compared with control conditions (SMD = −3.489 ± 1.551, 95% CI: −6.690 to −0.288, P = 0.034; Supplementary Table 4). However, substantial heterogeneity was observed among studies (I^2^ = 82.40%, P = 0.0034; SFig. 1D), indicating considerable variability in the magnitude of treatment effects. Therefore, although the pooled estimate suggests a favorable effect of GLP-1 RAs on MitoROS, the wide confidence interval, limited number of contributing studies, and high between-study heterogeneity reduce confidence in the precision and generalizability of this effect estimate.

### 3.4 Mitochondrial membrane potential (MMP)

Five studies contributed MMP data to the quantitative synthesis (Fig. 3B). Because some studies reported multiple eligible MMP outcomes, a total of seven effect sizes were included in the meta-analysis. Specifically, Liu et al. (2022) and Du et al. (2019) each contributed two effect sizes. The pooled analysis demonstrated no significant overall effect of GLP-1 receptor agonist treatment on MMP compared with control conditions (SMD = 0.997 ± 1.336, 95% CI: −1.678 to 3.672, P = 0.459; Supplementary Table 5). Heterogeneity was substantial (I^2^ = 89.98%, P < 0.0001; SFig. 1E).

To investigate potential sources of heterogeneity, outlier diagnostics were performed using a robust fit (Cauchy) procedure. This analysis identified both effect sizes reported by Liu et al. (2022) as statistical outliers. A sensitivity analysis excluding these outlying effect sizes was therefore conducted (Fig. 3C). Following exclusion of these statistically identified outlying effect sizes, the exploratory sensitivity analysis showed that GLP-1 RA treatment was associated with a significant increase in MMP (SMD = 3.145 ± 0.495, 95% CI: 2.147–4.144, P < 0.01**;** Supplementary Table 6), while heterogeneity decreased substantially (I^2^ = 19.10%, P = 0.2393; SFig. 1F).

Stratification of MMP outcomes by GLP-1 RA type in adipose tissue showed generally positive effects across exenatide-, lixisenatide-, and liraglutide-based interventions. However, apparent differences between agents should be interpreted with caution because agent type was confounded with study-specific experimental conditions and cell models, and the available evidence was derived from a limited number of studies (Fig. 3D; Supplementary Table 7).

## 4 Discussion

GLP-1 RAs were associated with significantly improved bioenergetic function and reduced MitoROS production in human-derived experimental models. In contrast, the primary meta-analysis did not demonstrate a significant effect on MMP, although an outlier-based sensitivity analysis showed a significant effect. Overall certainty of evidence was very low for the primary outcomes and low for the MMP sensitivity analysis, reflecting methodological limitations, small sample sizes, heterogeneity, and publication bias. Consequently, these findings should be interpreted as suggestive rather than definitive evidence of direct mitochondrial effects of GLP-1 RAs.

In small-sample preclinical meta-analyses, excluding studies based solely on risk-of-bias assessments may introduce greater instability than including them and appropriately downgrading certainty. Accordingly, all eligible studies were retained in the quantitative synthesis, and risk-of-bias assessments were incorporated into the modified GRADE framework rather than used as exclusion criteria.

Although substantial heterogeneity was observed across several analyses, this variability may reflect methodological differences between studies—including variation in cell models, disease contexts, GLP-1 RAs, exposure conditions, and mitochondrial outcome measures—rather than true biological disagreement. Because formal analyses such as meta-regression were not feasible given the limited number of studies, these potential sources of heterogeneity should be regarded as hypotheses rather than demonstrated explanations.

Experimental models varied widely and included neuronal systems (e.g., LUHMES dopaminergic-like neurons and primary neuronal cultures), metabolically active cell types (e.g., HepG2 hepatocytes, SGBS adipocytes, and human umbilical vein endothelial cells), and disease-relevant stress paradigms such as high-glucose exposure and inflammatory stimulation. These models differ substantially in baseline mitochondrial function, metabolic demand, and susceptibility to cellular stress, contributing to variability in the magnitude of treatment effects.

Heterogeneity was further amplified using diverse mitochondrial outcome measures. Studies assessed mitochondrial functional integrity through membrane potential measurements and cellular bioenergetics through oxygen consumption rate and ATP-related endpoints. These assays differ in sensitivity, temporal resolution, and mechanistic scope, limiting direct comparability across studies and contributing to statistical inconsistency. Nevertheless, the directions of effect were generally consistent, suggesting that variability reflected differences in experimental design and model systems rather than fundamentally contradictory biological findings.

Additional heterogeneity likely arose from differences among GLP-1 RAs and treatment paradigms. Included studies investigated exenatide/exendin-4, liraglutide, lixisenatide, semaglutide, and novel incretin analogs. Although these agents all activate the GLP-1 receptor, they differ in receptor affinity, intracellular signaling characteristics, pharmacokinetic stability, and experimental dosing strategies. Furthermore, treatment durations, concentrations, and intervention timing (preventive versus rescue designs) varied substantially across studies.

Despite this methodological diversity, the observed improvements in bioenergetics and reductions in MitoROS are biologically plausible and consistent with established GLP-1 receptor signaling pathways. Activation of the GLP-1 receptor stimulates intracellular cAMP signaling and downstream pathways involving protein kinase A (PKA), phosphoinositide 3-kinase (PI3K)/Akt, and AMP-activated protein kinase (AMPK), all of which have been implicated in mitochondrial regulation [36,37]. Previous experimental studies have suggested that GLP-1 receptor activation may influence multiple aspects of mitochondrial biology, including oxidative phosphorylation, ATP production, oxidative stress regulation, mitophagy, and mitochondrial biogenesis. In the present review, however, the synthesized outcomes were limited to mitochondrial bioenergetics, mitochondrial membrane potential, and mitochondrial reactive oxygen species. Consequently, our findings support direct effects of GLP-1 RAs on these measured aspects of mitochondrial function but do not directly establish effects on mitochondrial quality-control pathways [[19], [20], [21],^26^,^27^].

An important consideration when interpreting these findings is that the included studies primarily utilized human-derived cells, tissues, and ex vivo experimental models rather than whole-organism systems. This represents both a strength and a limitation. By isolating cellular responses to GLP-1 RAs, these models minimize confounding factors commonly encountered in clinical studies, including weight loss, changes in energy intake, alterations in insulin sensitivity, and other systemic metabolic adaptations. Consequently, the observed improvements in bioenergetics and reductions in MitoROS are more likely to reflect direct cellular and mitochondrial effects of GLP-1 receptor activation. However, these reductionist models cannot fully capture the complex physiological interactions that occur *in vivo* among tissues, organs, endocrine signaling pathways, and metabolic feedback networks. Therefore, while the present findings provide evidence supporting direct mitochondrial actions of GLP-1 RAs, their magnitude and translational relevance within whole-body physiology remain to be established.

Risk-of-bias assessment, quantitative synthesis, and GRADE certainty ratings were internally aligned. Outcomes that included studies at higher risk of bias were associated with lower certainty ratings, while downgrades for inconsistency, imprecision, and publication bias corresponded directly to statistical indicators such as I^2^ values, confidence interval width, and formal tests for small-study effects. Although several pooled analyses demonstrated large effect sizes, these findings should be interpreted with caution given the preclinical nature of the included studies, the lack of independence among some outcome measures, and the potential for effect inflation inherent in mechanistic assays.

Although the included studies support the presence of mitochondrial effects following GLP-1 RA exposure in isolated human-derived experimental systems, the intracellular signaling mechanisms underlying these effects remain incompletely characterized. A limited number of studies implicated pathways involving SIRT1, PINK1/Parkin, Akt, and ER-mitochondrial signaling [14,20,21], but comprehensive mechanistic validation using receptor-specific antagonism, genetic knockdown, or knockout approaches was uncommon. Consequently, the term "direct effects” in this review refers to effects observed independently of systemic physiological adaptation rather than definitive evidence of direct intracellular mitochondrial targeting.

Collectively, these findings provide preliminary evidence that GLP-1 RAs may directly influence mitochondrial function in human-derived experimental models, particularly through improvements in bioenergetic measures and reductions in mitochondrial oxidative stress. However, substantial heterogeneity, methodological limitations, small sample sizes, publication bias, and the low-to-very-low certainty of the evidence limit confidence in these findings.

Future studies should employ standardized mitochondrial outcome measures, improved methodological reporting, mechanistic validation, and independent replication to determine whether these observed cellular effects are reproducible and relevant to whole-body physiology and clinical outcomes. In addition, future investigations should prioritize the use of primary human cells, tissue-derived models, and other physiologically relevant experimental systems to improve the translational applicability of mechanistic findings. Particular attention should be given to reducing reliance on immortalized cell lines, including SH-SY5Y neuroblastoma cells, which may exhibit metabolic and bioenergetic characteristics that differ from those of primary human tissues. Given the substantial contribution of a limited number of research groups and experimental platforms to the current evidence base, replication by independent, unaffiliated research groups will be important for establishing the robustness, generalizability, and translational relevance of the reported mitochondrial effects before strong translational inferences can be made.

### 4.1 Limitations

Several limitations should be considered when interpreting the findings of this systematic review and meta-analysis. First, the number of eligible mechanistic studies contributing to each mitochondrial outcome was limited, precluding meaningful sensitivity analyses based on overall risk-of-bias category. Consequently, all eligible studies were retained in the quantitative synthesis, and methodological limitations were incorporated into the modified GRADE assessment rather than used as exclusion criteria. Accordingly, certainty ratings reflect the quality of the underlying evidence and should be considered alongside the observed direction and magnitude of the pooled effects.

Second, several studies reported multiple conceptually related bioenergetic outcomes, including ATP-linked oxygen consumption, basal respiration, maximal respiration, and ATP production. To maximize use of the available evidence, these outcomes were analyzed as separate effect sizes within the bioenergetics meta-analysis. However, because within-study correlations among these measures were not reported, multilevel or multivariate meta-analysis was not feasible, and the effect sizes were therefore treated as statistically independent. This approach may have increased the relative weight of studies reporting multiple bioenergetic endpoints and overestimated the statistical precision of the pooled bioenergetic estimates. Consequently, the magnitude and precision of these pooled estimates should be interpreted cautiously.

Third, formal assessments of publication bias should also be interpreted with caution because most meta-analyses included fewer than 10 studies. Funnel plots and Egger's and Begg's tests have limited statistical power under these circumstances and therefore cannot reliably exclude publication bias or small-study effects.

Fourth, the subgroup analyses by tissue type and GLP-1 receptor agonist class were exploratory rather than prespecified and were conducted to investigate potential sources of heterogeneity identified after study selection. Given the limited number of contributing studies, these analyses should be regarded as hypothesis-generating rather than confirmatory.

Furthermore, the exclusive reliance on in vitro and ex vivo experimental models limits the ability to account for systemic physiological processes, inter-organ communication, endocrine regulation, and tissue crosstalk that influence mitochondrial function *in vivo*. Accordingly, the observed mitochondrial effects should be interpreted as mechanistic evidence obtained under controlled experimental conditions rather than direct evidence of organism-level physiological responses.

An additional limitation is that several included studies employed immortalized or transformed human cell lines, particularly SH-SY5Y neuroblastoma cells, rather than primary human cells or tissue-derived models. Although these systems provide valuable mechanistic insight and are widely used in mitochondrial research, they may exhibit metabolic characteristics that differ from those of primary human tissues, including altered mitochondrial function, substrate utilization, and cellular bioenergetics [38]. Consequently, the observed effects on mitochondrial respiration, ATP production, mitochondrial membrane potential, and oxidative stress may not fully reflect responses occurring in native human tissues, limiting the translational applicability of these findings.

The current evidence base is also limited by incomplete mechanistic validation. Most included studies relied primarily on pharmacological exposure to GLP-1 receptor agonists without comprehensive confirmation of receptor specificity. Although several investigations incorporated pathway-focused experiments, including Parkin RNA silencing and assessment of PINK1/Parkin-, SIRT1-, and Akt-related signaling, functional genetic approaches such as GLP-1 receptor knockdown, receptor knockout, or targeted manipulation of downstream mitochondrial regulators were uncommon. Consequently, the causal mechanisms linking GLP-1 receptor activation to the observed mitochondrial effects remain incompletely defined and require further investigation.

Relatedly, GLP-1 receptor agonists exert broad cytoprotective effects, including reductions in oxidative stress, inflammation, and apoptosis. Therefore, improvements in mitochondrial outcomes do not necessarily indicate that mitochondria represent the primary intracellular target of GLP-1 receptor agonist action. Rather, some observed improvements in mitochondrial respiration, membrane potential, and oxidative stress may reflect secondary consequences of enhanced overall cellular health and stress resilience. Because relatively few studies have incorporated mechanistic experiments that can establish causal relationships among receptor activation, mitochondrial changes, and downstream cellular responses, the specificity and directionality of these interactions remain uncertain.

Finally, no validated reporting-quality instrument currently exists specifically for human-derived mechanistic in vitro studies. Consequently, general reporting principles derived from the ARRIVE guidelines were used to inform assessment of methodological transparency and reporting completeness, although these guidelines have not been formally validated for this experimental context.

## 5 Conclusion

This systematic review and meta-analysis identified evidence consistent with potential direct mitochondrial effects of GLP-1 RAs in human-derived cellular and tissue models. However, substantial heterogeneity across studies limits the certainty and interpretability of these findings. Although the primary analysis of mitochondrial membrane potential did not demonstrate a significant effect, an outlier-based sensitivity analysis excluding statistically identified outliers suggested a possible improvement in mitochondrial membrane potential; however, this finding should be interpreted with caution.

Substantial heterogeneity was observed across studies, potentially influenced by differences in experimental models, disease contexts, mitochondrial outcome measures, treatment paradigms, and GLP-1 RA types. Although many individual effect estimates favored GLP-1 RA treatment, the magnitude of effects varied considerably across studies. The certainty of the evidence remained low to very low due to methodological limitations, small sample sizes, publication bias, and the preclinical nature of the included studies.

## Data availability and resource availability statement

The datasets generated and analyzed during the current study are available from the corresponding author upon reasonable request.

## Artificial intelligence

***Artificial intelligence*** technology was used to improve readability and language (Grammarly). The graphical abstract was generated using ChatGPT (OpenAI), BioRender, and Preview based on detailed author-provided instructions. The authors determined the structure, selected all scientific elements, reviewed multiple iterations, corrected inaccuracies, and approved the final version.

## Funding disclosure

This project was supported by Institutional funds. Its contents are solely the responsibility of the authors and do not necessarily represent the official views of the School of Veterinary Medicine.

## CRediT authorship contribution statement

**Zoe Lee Greenblatt:** Data curation, Formal analysis, Investigation, Methodology, Validation, Writing – original draft. **Atena Tork:** Data curation, Investigation, Methodology, Validation. **Ivy Cruz:** Data curation, Investigation, Methodology, Validation. **Erik D. Fausak:** Data curation, Formal analysis, Investigation, Methodology, Resources, Supervision, Validation, Writing – review & editing. **Huy-an Tran:** Investigation, Methodology. **Mahima Krovidy:** Investigation, Methodology. **Cecilia Giulivi:** Conceptualization, Data curation, Formal analysis, Investigation, Methodology, Project administration, Supervision, Validation, Visualization, Writing – review & editing.

## Financial or other conflicts of interest

All authors have disclosed any financial or other interests related to the submitted work that could impact the authors' objectivity or influence the article's content. No financial or non-financial competing interests were identified that could compromise the objectivity, integrity, or value of this publication by influencing the authors' judgment and actions in data presentation, analysis, and interpretation. C.G. serves as an Editorial Board Member of Scientific Reports. She has received compensation as a Field Chief Editor for Frontiers in Molecular Biosciences and honoraria for participating in NIH peer review meetings.
