# Supplementary material for: Direct effects of glucagon-like Peptide-1 receptor agonists on mitochondrial function in human-derived in vitro models: A systematic review and meta-analysis
Source: Metabol Open. 2026 Jul 2;31:100484. doi: 10.1016/j.metop.2026.100484 (PMC13351396; doi:10.1016/j.metop.2026.100484)
Supplement: Multimedia component 1 [file mmc1.pdf]

Supplementary Information for "Direct Effects of Glucagon-Like Peptide-1 Receptor Agonists on Mitochondrial Function in Human-Derived In Vitro Models: A Systematic Review and Meta-Analysis" by Lee Greenblatt et al. (2026)

SECTION	PAGE
**SUPPLEMENTARY METHODS**	
PRISMA-S SEARCH STRATEGY	2
**SUPPLEMENTAL FIGURES**	
SUPPLEMENTAL FIGURE 1.<br>FUNNEL PLOTS EVALUATING POTENTIAL PUBLICATION BIAS IN STUDIES OF MITOCHONDRIAL OUTCOMES FOLLOWING GLP-1 RECEPTOR AGONIST TREATMENT	6
**SUPPLEMENTARY TABLES**	
SUPPLEMENTAL TABLE 1:<br>BIOENERGETICS META-ANALYSIS (ALL STUDIES)	7
SUPPLEMENTAL TABLE 2:<br>BIOENERGETICS META-ANALYSIS (ADIPOSE TISSUE ONLY)	9
SUPPLEMENTAL TABLE 3:<br>BIOENERGETICS META-ANALYSIS IN ADIPOSE TISSUE AND STRATIFIED BY DRUG TYPE	11
SUPPLEMENTAL TABLE 4:<br>MITOROS META-ANALYSIS	13
SUPPLEMENTAL TABLE 5:<br>MMP META-ANALYSIS	14
SUPPLEMENTAL TABLE 6:<br>MMP META-ANALYSIS EXCLUDING LIU ET AL (2022)	16
SUPPLEMENTAL TABLE 7:<br>MMP META-ANALYSIS STRATIFIED BY DRUG TYPE	17

PRISMA-S Template (based on v1.0 retrieved from https://osf.io/2ybwn/)

(Literature and information being sought) Mitochondrial involvement with Glucagon-Like Peptide-1 Receptor Agonists (GLP-1 RAs) Is there solid evidence that mitochondrial activity is associated with GLP-1 RAs?

Databases and Interfaces Searched:

Database	Interface	Date Coverage	Date Searched
Medline (included products: Medline, in process citations, “ahead of print” citations, out-of-scope citations, journals indexing prior to Medline inclusion, pre-1966 citations, PubMed Central, author manuscripts NIH funding, NCBI Bookshelf)	Pubmed	2010-2026	31 March 2026
Chemical Abstracts	Scifinder	1996-2026	31 March 2026
Scopus	Elsevier or Scopus (Elsevier) or Embase	2006-2026	31 March 2026
Embase	Elsevier or Embase (Elsevier) OR Embase	2009-2026	30 March 2026

Simultaneous Searches: None performed
Item 2: Other Online Resources (As Needed): N/A
Manual Searching (searching relevant journals’ Table of Contents): N/A

Citation Searching And Text Analysis:
Process: Searching was performed using keywords and utilizing database indexing by all authors and a content expert (CG)
Contacts (Researchers contacted for additional information): N/A
Additional Methodologies Not Listed Above: N/A

Limits and Restrictions
Date and Time Period: Not restricted
Language: English
Publication status: Published/peer reviewed
Species Included: Human
Study Design: *ex vivo/in vitro*, prospective experimental studies, Cohort, Case-control, cross-sectional
Database Subset: N/A
Pre-specified cut-off or saturation point for results: N/A
Other Restrictions: N/A

Search Filters:

Database	Interface	Search Filters Applied
Medline	Pubmed	Exclude: Review Include: Human
Scifinder	Scifinder	Exclude: Review, clinical trial, commentary, editorial, report<br>Include: Human, English
Scopus	Scopus	Exclude:review, book chapter, book, short survey editorial,<br>note, conference paper, retracted, letter, erratum<br>Limit to: English
Embase	Elsevier or Embase (Elsevier) OR Embase	Limited to: Article and Humans

Full Search Strategy:
Search Database: PubMed

Search ID	Terms	Results
#1	**"GLP-1 agonist"[tiab:~5] OR Glucagon-like peptide-1 receptor agonists OR "GLP-1 drugs"**	**18,124**
#2	**"Mitochondrial function" OR "mitochondrial dysfunction"**	**70,587**
#3	**#1 AND #2**	**186**
#4	**#3 AND Humans[Filter] AND English [filter] NOT Review[Filter]**	**29**
**Copy and paste**	**((("Mitochondrial function"[All Fields] OR "mitochondrial dysfunction"[All Fields]) AND ("GLP-1 agonist"[Title/Abstract:~5] OR ("glucagon like peptide 1 receptor agonists"[Pharmacological Action] OR "glucagon like peptide 1 receptor agonists"[Supplementary Concept] OR "glucagon like peptide 1 receptor agonists"[All Fields] OR "glucagon like peptide 1 receptor agonists"[All Fields] OR "glucagon like peptide 1 receptor agonists"[MeSH Terms] OR ("glucagon like"[All Fields] AND "peptide 1"[All Fields] AND "receptor"[All Fields] AND "agonists"[All Fields])) OR "GLP-1 drugs"[All Fields])) NOT "review"[Publication Type]) AND (humans[Filter]) AND (English [Filter])**	**29**

Search Database: Scifinder

Search ID	Terms	Results
#1	(GLP-1 receptor agonists or GLP-1 RA or Glucagon-like peptide-1 receptor agonists)	883,528
#2	("Mitochondrial Dysfunction" OR "Mitochondrial Function")	84,488
#3	#1 and #2 AND Human [subject] AND English [filte NOT filters applied [Review, clinical trial, commentary, editorial, report]	162
Copy and Paste	((GLP-1 receptor agonists or GLP-1 RA or Glucagon-like peptide-1 receptor agonists) AND ("Mitochondrial Dysfunction" OR "Mitochondrial Function"))	162

Search Database: Scopus

Search ID	Terms	Results
#1	GLP-1 W/5 agonist OR Glucagon-like peptide-1 receptor agonists OR "GLP-1 drugs"	32,064
#2	("Mitochondrial Dysfunction" OR "Mitochondrial Function")	466,562
#3	#1 and #2 LIMIT to Human [subject] AND English [filter] NOT filters applied [review, book chapter, book, short survey editorial, note, conference paper, retracted, letter, erratum]	420
Copy and Paste	(GLP-1 W/5 agonist OR Glucagon-like peptide-1 receptor agonists OR "GLP-1 drugs") AND ("Mitochondrial Dysfunction" OR "Mitochondrial Function") AND (EXCLUDE (DOCTYPE , "bk") OR EXCLUDE (DOCTYPE , "sh") OR EXCLUDE (DOCTYPE , "ed") OR EXCLUDE (DOCTYPE , "no") OR EXCLUDE (DOCTYPE , "cp") OR EXCLUDE (DOCTYPE , "tb") OR EXCLUDE (DOCTYPE , "le") OR EXCLUDE (DOCTYPE , "er") OR EXCLUDE (DOCTYPE , "re") OR EXCLUDE (DOCTYPE , "ch")) AND (LIMIT-TO (LANGUAGE , "English")) AND (LIMIT-TO (EXACTKEYWORD , "Humans"))	420

Search Database: Embase

Search ID	Terms	Results
#1	'glucagon like peptide 1 receptor agonist'/exp OR 'glucagon like peptide 1 receptor agonist'	75,574
#2	‘Mitochondrial AND Dysfunction’	86,068
#3	‘Mitochondrial AND Function’	151,716
#4	#2 OR #3	195, 256
#5	#1 AND #4 Limit to Article[Filter] and Humans [Filter]	171
Copy and paste	(('glucagon like peptide 1 receptor agonist’/exp OR ‘glucagon like peptide 1 receptor agonist’) AND (‘Mitochondrial AND Dysfunction’ OR ‘Mitochondrial AND Function’))	171

Updates: N/A

Search Designers: Undergraduate (ZLG), Librarian [EF], and content expert (CG) built the original search strategy, and all other authors helped translate it to other databases. All records were deduplicated with Covidence (Australia).

Peer Review: Reviewed by CG

Records Screened:

Total Records	Total Records after deduplication	Deduplication software/methodology
782	526	Covidence

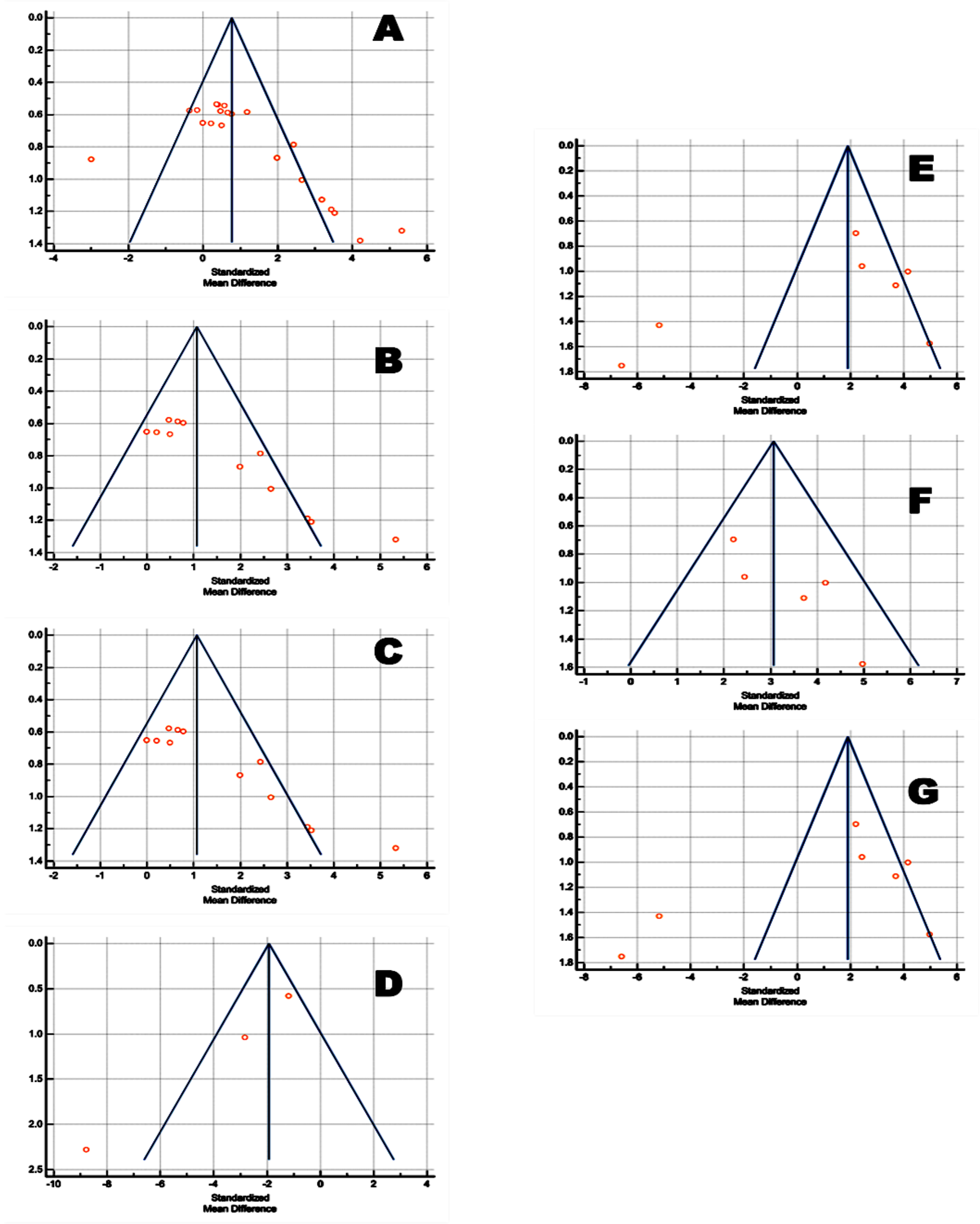

Supplemental Figure 1. Funnel plots evaluating potential publication bias in studies of mitochondrial outcomes following GLP-1 receptor agonist treatment. (A) Funnel plot for all bioenergetic outcomes. (B) Funnel plot for bioenergetic outcomes restricted to adipose tissue-derived models. (C) Funnel plot for adipose tissue bioenergetic outcomes stratified by GLP-1 receptor agonist type. (D) Funnel plot for mitochondrial reactive oxygen species (MitoROS) outcomes. (E) Funnel plot for mitochondrial membrane potential (MMP) outcomes. (F) Funnel plot for MMP outcomes following exclusion of Liu et al. (2022). (G) Funnel plot for MMP outcomes stratified by GLP-1 receptor agonist type. Each point represents an individual effect

size, and the vertical line indicates the pooled effect estimate. Symmetry around the pooled effect estimate was assessed visually and supplemented by Egger's regression test and Begg's rank correlation test.

SUPPLEMENTAL TABLE 1: BIOENERGETICS META-ANALYSIS (ALL STUDIES)

Variable for studies	Study
1. Intervention groups	
Variable for number of cases	GLP_1RA_N_
Variable for mean	GLP_1RA_mean_
Variable for SD	GLP_1RA_SD_
2. Control groups	
Variable for number of cases	CTL_N_
Variable for mean	CTL_mean_
Variable for SD	CTL_SD_

Study	N1	N2	Total	SMD	SE	95% CI	t	P	Weight (%)	
									Fixed	Random
Igoillo-esteve, 2020 (C)	6	6	12	0.394	0.539	-0.806 to 1.595			7.21	5.09
Igoillo-esteve, 2020 (A)	6	6	12	0.572	0.545	-0.643 to 1.788			7.04	5.06
Igoillo-esteve, 2020 (B)	6	6	12	0.369	0.538	-0.830 to 1.568			7.23	5.09
Goralska, 2017 (E)	5	5	10	0.776	0.597	-0.600 to 2.152			5.88	4.88
Goralska, 2017 (A)	5	5	10	0.664	0.590	-0.696 to 2.024			6.01	4.90
Goralska, 2017 (B)	5	5	10	2.428	0.788	0.611 to 4.245			3.37	4.19
Goralska, 2017 (C)	5	5	10	0.469	0.580	-0.870 to 1.807			6.21	4.94
Goralska, 2017 (D)	5	5	10	5.329	1.321	2.282 to 8.375			1.20	2.62
Vaittinen, 2023 (G)	3	3	6	0.000	0.651	-1.809 to 1.809			4.93	4.68
Vaittinen, 2023 (I)	3	3	6	0.000	0.651	-1.809 to 1.809			4.93	4.68
Vaittinen, 2023 (H)	3	3	6	0.500	0.667	-1.352 to 2.353			4.70	4.62
Qi, 2022 (B)	6	6	12	1.191	0.586	-0.114 to 2.496			6.10	4.92
Yu, 2019 (B)	3	3	6	4.217	1.381	0.384 to 8.051			1.10	2.49
Panagaki, 2023 (C)	5	5	10	-0.354	0.576	-1.683 to 0.975			6.30	4.95
Panagaki, 2023 (A)	5	5	10	-2.991	0.879	-5.018 to -0.963			2.71	3.88

Yu, 2019 (A)	3	3	6	3.192	1.128	0.0587 to 6.324			1.64	3.11
Panagaki, 2023 (B)	5	5	10	-0.156	0.572	-1.475 to 1.163			6.40	4.97
Yu, 2019 (C)	3	3	6	3.192	1.128	0.0587 to 6.324			1.64	3.11
Vaittinen, 2023 (B)	3	3	6	2.660	1.007	-0.136 to 5.455			2.06	3.47
Vaittinen, 2023 (F)	3	3	6	3.443	1.188	0.143 to 6.742			1.48	2.95
Vaittinen, 2023 (D)	3	3	6	3.532	1.210	0.173 to 6.892			1.43	2.89
Vaittinen, 2023 (A)	3	3	6	1.995	0.869	-0.419 to 4.409			2.77	3.91
Vaittinen, 2023 (E)	3	3	6	0.213	0.654	-1.604 to 2.030			4.89	4.67
Vaittinen, 2023 (C)	3	3	6	1.995	0.869	-0.419 to 4.409			2.77	3.91
Total (fixed effects)	100	100	200	0.767	0.145	0.482 to 1.053	5.303	<0.001	100.00	100.00
Total (random effects)	100	100	200	1.109	0.281	0.556 to 1.662	3.952	<0.001	100.00	100.00

Test for heterogeneity

Q	79.9265
DF	23
Significance level	P < 0.0001
I^2 (inconsistency)	71.22%
95% CI for I^2	56.58 to 80.93

Publication bias

Egger's test	
Intercept	4.6130
95% CI	2.5153 to 6.7107
Significance level	P = 0.0002
Begg's test	
Kendall's Tau	0.5971
Significance level	P < 0.0001

SUPPLEMENTAL TABLE 2: BIOENERGETICS META-ANALYSIS (ADIPOSE TISSUE ONLY)

Variable for studies	Study
1. Intervention groups	
Variable for number of cases	GLP_1RA_N_
Variable for mean	GLP_1RA_mean_
Variable for SD	GLP_1RA_SD_
2. Control groups	
Variable for number of cases	CTL_N_
Variable for mean	CTL_mean_

Variable for SD	CTL_SD_

Study	N1	N2	Total	SMD	SE	95% CI	t	P	Weight (%)	
									Fixed	Random
Goralska, 2017 (E)	5	5	10	0.776	0.597	-0.600 to 2.152			11.17	8.72
Goralska, 2017 (A)	5	5	10	0.664	0.590	-0.696 to 2.024			11.43	8.77
Goralska, 2017 (B)	5	5	10	2.428	0.788	0.611 to 4.245			6.40	7.29
Goralska, 2017 (C)	5	5	10	0.469	0.580	-0.870 to 1.807			11.80	8.84
Goralska, 2017 (D)	5	5	10	5.329	1.321	2.282 to 8.375			2.28	4.30
Vaittinen, 2023 (G)	3	3	6	0.000	0.651	-1.809 to 1.809			9.37	8.30
Vaittinen, 2023 (I)	3	3	6	0.000	0.651	-1.809 to 1.809			9.37	8.30
Vaittinen, 2023 (H)	3	3	6	0.500	0.667	-1.352 to 2.353			8.93	8.18
Vaittinen, 2023 (B)	3	3	6	2.660	1.007	-0.136 to 5.455			3.92	5.87
Vaittinen, 2023 (F)	3	3	6	3.443	1.188	0.143 to 6.742			2.82	4.90
Vaittinen, 2023 (D)	3	3	6	3.532	1.210	0.173 to 6.892			2.72	4.79
Vaittinen, 2023 (A)	3	3	6	1.995	0.869	-0.419 to 4.409			5.26	6.73
Vaittinen, 2023 (E)	3	3	6	0.213	0.654	-1.604 to 2.030			9.29	8.28
Vaittinen, 2023 (C)	3	3	6	1.995	0.869	-0.419 to 4.409			5.26	6.73
Total (fixed effects)	52	52	104	1.066	0.199	0.671 to 1.462	5.347	<0.001	100.00	100.00
Total (random effects)	52	52	104	1.394	0.343	0.713 to 2.075	4.061	<0.001	100.00	100.00

Test for heterogeneity

Q	35.8725
DF	13
Significance level	P = 0.0006
I^2 (inconsistency)	63.76%
95% CI for I^2	35.71 to 79.57

Publication bias

Egger's test	
Intercept	5.9074

95% CI	4.2739 to 7.5408
Significance level	P < 0.0001
Begg's test	
Kendall's Tau	0.6854
Significance level	P = 0.0006

SUPPLEMENTAL TABLE 3: BIOENERGETICS META-ANALYSIS IN ADIPOSE TISSUE AND STRATIFIED BY DRUG TYPE

Variable for studies	GLP_1RA_studied
1. Intervention groups	
Variable for number of cases	GLP_1RA_N_
Variable for mean	GLP_1RA_mean_
Variable for SD	GLP_1RA_SD_
2. Control groups	
Variable for number of cases	CTL_N_
Variable for mean	CTL_mean_
Variable for SD	CTL_SD_

Study	N1	N2	Total	SMD	SE	95% CI	t	P	Weight (%)	
									Fixed	Random
Exendin-4	5	5	10	0.776	0.597	-0.600 to 2.152			11.17	8.72
Exendin-4	5	5	10	0.664	0.590	-0.696 to 2.024			11.43	8.77
Exendin-4	5	5	10	2.428	0.788	0.611 to 4.245			6.40	7.29
Exendin-4	5	5	10	0.469	0.580	-0.870 to 1.807			11.80	8.84
Exendin-4	5	5	10	5.329	1.321	2.282 to 8.375			2.28	4.30
Liraglitude+TNFalpha	3	3	6	0.000	0.651	-1.809 to 1.809			9.37	8.30
Liraglitude+TNFalpha	3	3	6	0.000	0.651	-1.809 to 1.809			9.37	8.30
Liraglitude+TNFalpha	3	3	6	0.500	0.667	-1.352 to 2.353			8.93	8.18
Liraglutide-LT	3	3	6	2.660	1.007	-0.136 to 5.455			3.92	5.87
Liraglutide-LT	3	3	6	3.443	1.188	0.143 to 6.742			2.82	4.90

Liraglutide-LT	3	3	6	3.532	1.210	0.173 to 6.892			2.72	4.79
Liraglutide-ST	3	3	6	1.995	0.869	-0.419 to 4.409			5.26	6.73
Liraglutide-ST	3	3	6	0.213	0.654	-1.604 to 2.030			9.29	8.28
Liraglutide-ST	3	3	6	1.995	0.869	-0.419 to 4.409			5.26	6.73
Total (fixed effects)	52	52	104	1.066	0.199	0.671 to 1.462	5.347	<0.001	100.00	100.00
Total (random effects)	52	52	104	1.394	0.343	0.713 to 2.075	4.061	<0.001	100.00	100.00

Test for heterogeneity

Q	35.8725
DF	13
Significance level	P = 0.0006
I^2 (inconsistency)	63.76%
95% CI for I^2	35.71 to 79.57

Publication bias

Egger's test	
Intercept	5.9074
95% CI	4.2739 to 7.5408
Significance level	P < 0.0001
Begg's test	
Kendall's Tau	0.6854
Significance level	P = 0.0006

SUPPLEMENTAL TABLE 4: MITOROS META-ANALYSIS

Variable for studies	Study
1. Intervention groups	
Variable for number of cases	GLP_1RA_N_ GLP-1RA (N)
Variable for mean	GLP_1RA_mean_ GLP-1RA (mean)
Variable for SD	GLP_1RA_SD_ GLP-1RA (SD)
2. Control groups	
Variable for number of cases	CTL_N_ CTL (N)
Variable for mean	CTL_mean_ CTL (mean)
Variable for SD	CTL_SD_ CTL (SD)

Study	N1	N2	Total	SMD	SE	95% CI	t	P	Weight (%)	
									Fixed	Random
Pandey, 2023 (B)	4	4	8	-8.783	2.280	-14.362 to -3.204			4.77	22.46
Qi, 2022 (C)	6	6	12	-1.189	0.585	-2.494 to 0.115			72.40	41.09
Zhang, 2022 (B)	3	3	6	-2.820	1.043	-5.714 to 0.0750			22.83	36.45
Total (fixed effects)	13	13	26	-1.924	0.498	-2.952 to -0.896	-3.863	0.001	100.00	100.00
Total (random effects)	13	13	26	-3.489	1.551	-6.690 to -0.288	-2.250	0.034	100.00	100.00

Test for heterogeneity

Q	11.3616
DF	2
Significance level	P = 0.0034
I^2 (inconsistency)	82.40%
95% CI for I^2	45.95 to 94.27

Publication bias

Egger's test	
Intercept	-4.3086
95% CI	-9.6237 to 1.0065
Significance level	P = 0.0616
Begg's test	
Kendall's Tau	-1.0000
Significance level	P = 0.1172

SUPPLEMENTAL TABLE 5: MMP META-ANALYSIS

Variable for studies	Study
1. Intervention groups	
Variable for number of cases	GLP_1RA_N_
Variable for mean	GLP_1RA_mean_
Variable for SD	GLP_1RA_SD_
2. Control groups	
Variable for number of cases	CTL_N_
Variable for mean	CTL_mean_
Variable for SD	CTL_SD_

Study	N1	N2	Total	SMD	SE	95% CI	t	P	Weight (%)	
									Fixed	Random
Jo, 2021	3	3	6	2.453	0.962	-0.218 to 5.125			17.58	15.04
Pandey, 2023 (A)	4	4	8	3.715	1.114	0.991 to 6.440			13.13	14.65
Liu 2022 (A)	4	4	8	-5.163	1.429	-8.661 to -1.665			7.97	13.75
Liu 2022 (B)	4	4	8	-6.575	1.755	-10.869 to -2.281			5.29	12.73
Zhang, 2022 (A)	3	3	6	4.980	1.578	0.598 to 9.362			6.53	13.29
Du, 2019 (A)	6	6	12	2.210	0.698	0.654 to 3.765			33.41	15.62
Du, 2019 (B)	6	6	12	4.178	1.006	1.938 to 6.419			16.10	14.93
Total (fixed effects)	30	30	60	1.896	0.403	1.089 to 2.704	4.700	<0.001	100.00	100.00
Total (random effects)	30	30	60	0.997	1.336	-1.678 to 3.672	0.746	0.459	100.00	100.00

Test for heterogeneity

Q	59.8659
DF	6
Significance level	P < 0.0001
I^2 (inconsistency)	89.98%
95% CI for I^2	81.90 to 94.45

Publication bias

Egger's test	
Intercept	-4.7547
95% CI	-14.7870 to 5.2776
Significance level	P = 0.2775

Begg's test	
Kendall's Tau	-0.04762
Significance level	P = 0.8806

SUPPLEMENTAL TABLE 6: MMP META-ANALYSIS EXCLUDING LIU ET AL (2022)

Variable for studies	Study
1. Intervention groups	
Variable for number of cases	GLP_1RA_N_
Variable for mean	GLP_1RA_mean_
Variable for SD	GLP_1RA_SD_
2. Control groups	
Variable for number of cases	CTL_N_
Variable for mean	CTL_mean_
Variable for SD	CTL_SD_

Study	N1	N2	Total	SMD	SE	95% CI	t	P	Weight (%)	
									Fixed	Random
Jo, 2021	3	3	6	2.453	0.962	-0.218 to 5.125			20.27	21.05
Pandey, 2023 (A)	4	4	8	3.715	1.114	0.991 to 6.440			15.13	16.57
Zhang, 2022 (A)	3	3	6	4.980	1.578	0.598 to 9.362			7.53	8.97
Du, 2019 (A)	6	6	12	2.210	0.698	0.654 to 3.765			38.51	33.80
Du, 2019 (B)	6	6	12	4.178	1.006	1.938 to 6.419			18.56	19.61
Total (fixed effects)	22	22	44	3.061	0.433	2.187 to 3.935	7.066	<0.001	100.00	100.00
Total (random effects)	22	22	44	3.145	0.495	2.147 to 4.144	6.357	<0.001	100.00	100.00

Test for heterogeneity

Q	4.9447
DF	4
Significance level	P = 0.2930
I^2 (inconsistency)	19.10%
95% CI for I^2	0.00 to 84.16

Publication bias

Egger's test	
Intercept	3.4017
95% CI	-0.3806 to 7.1840
Significance level	P = 0.0645
Begg's test	
Kendall's Tau	0.8000
Significance level	P = 0.0500

SUPPLEMENTAL TABLE 7: MMP META-ANALYSIS STRATIFIED BY DRUG TYPE

Variable for studies	GLP_1RA_stuided<br>GLP-1RA stuided
1. Intervention groups	
Variable for number of cases	GLP_1RA_N_<br>GLP-1RA (N)
Variable for mean	GLP_1RA_mean_<br>GLP-1RA (mean)
Variable for SD	GLP_1RA_SD_<br>GLP-1RA (SD)
2. Control groups	
Variable for number of cases	CTL_N_<br>CTL (N)
Variable for mean	CTL_mean_<br>CTL (mean)
Variable for SD	CTL_SD_<br>CTL (SD)

Study	N1	N2	Total	SMD	SE	95% CI	t	P	Weight (%)	
									Fixed	Random
Exenatide	3	3	6	2.453	0.962	-0.218 to 5.125			17.58	15.04
Exendin-4	4	4	8	3.715	1.114	0.991 to 6.440			13.13	14.65
Liraglutide	4	4	8	-5.163	1.429	-8.661 to -1.665			7.97	13.75
Liraglutide	4	4	8	-6.575	1.755	-10.869 to -2.281			5.29	12.73
Liraglutide	3	3	6	4.980	1.578	0.598 to 9.362			6.53	13.29
Lixisenatide	6	6	12	2.210	0.698	0.654 to 3.765			33.41	15.62
Lixisenatide	6	6	12	4.178	1.006	1.938 to 6.419			16.10	14.93
Total (fixed effects)	30	30	60	1.896	0.403	1.089 to 2.704	4.700	<0.001	100.00	100.00
Total (random effects)	30	30	60	0.997	1.336	-1.678 to 3.672	0.746	0.459	100.00	100.00

Test for heterogeneity

Q	59.8659
DF	6
Significance level	P < 0.0001
I^2 (inconsistency)	89.98%
95% CI for I^2	81.90 to 94.45

Publication bias

Egger's test

Intercept	-4.7547
95% CI	-14.7870 to 5.2776
Significance level	P = 0.2775
Begg's test	
Kendall's Tau	-0.04762
Significance level	P = 0.8806
